# Advances in Micro- and Nanomechanics

**DOI:** 10.3390/nano12244433

**Published:** 2022-12-13

**Authors:** Victor A. Eremeyev

**Affiliations:** Department of Civil and Environmental Engineering and Architecture (DICAAR), University of Cagliari, Via Marengo, 2, 09123 Cagliari, Italy; victor.eremeev@unica.it or eremeyev.victor@gmail.com

Recent advances in technologies of design, manufacturing and further studies of new materials and structures result in an essential extension of classic models of continuum and structural mechanics. For example, nowadays it is well established that material properties at small scales could be size-dependent. In the literature, various approaches were proposed for studying such phenomena. It is worth mentioning stress and strain gradient elasticity, surface elasticity, media with internal degrees of freedom, and nonlocal continua among others. These models can successfully describe various sized effects.

Another direction in the mechanics of materials, close-related to the aforementioned enhancements, addresses theoretical and experimental studies of material properties for media with complex internal microstructure. In particular, the great interest is the determination of effective properties of new composite materials and further analysis of their dependence on the microstructure.

Let us also note that at micro- and nano-scales one can observe a more rich picture of electromechanical couplings. The latter may play an important role in material response. For example, so-called flexoeffects relate electric polarization or magnetization to gradients of strains. So both flexoelectricity and flexomagneticity bring us another example of strain gradient models. Being sometimes even negligible at the macro-scale, these properties may be dominant at the nanoscale. This gives a possibility to use such materials as elements of MEMS and NEMS, such as energy harvesters, sensors, and actuators.

This special issue “Advances in Micro- and Nanomechanics” collects several papers that have presented theoretical, numerical, and experimental studies of materials and structures at small scales. It is rather natural to expect new phenomena in nanometer-sized thin-walled structures such as nanowires and nanofilms.

The new model of a nanowire embedded into an elastic substrate was proposed in [1]. Here surface energy was taken into account as in the Gurtin–Murdoch surface elasticity as well as a nonlocality according to the strain gradient approach. For the derivation of the governing equations, the virtual force technique was applied.

Experimental studies of thin films were presented in [2,3,4]. Here, films were produced with magnetron sputtering and further analyzed using various techniques such as atomic force microscopy and nanoindentation. As a result, microstructural, nanomechanical, and tribological properties were discussed in more detail. The residual stress-driven technique was applied to the determination of Young’s modulus of nanofilms in [5]. Here authors proposed a new relatively simple approach based on the consideration of deformations of bilayer cantilevers. The analysis of the thermal stability and hardness of nanocrystalline Ni thin films was given in [6]. Here it was shown that the addition of cysteine results in improved hardness of films.

Properties of nanoparticles and related composites were investigated in [7,8]. Fracture strength and local hardness of spherical particles made of B${}_{4}$C and TiC were estimated in [7]. In [8], nanoparticles of NiO/C applied for the manufacturing of nanocomposites for supercapacitors were investigated using X-ray diffraction and other techniques.

Biomechanical studies of coatings were discussed in [9]. Here authors discussed the mechanical properties, microstructure, and composition of enamel and dentine at the initial stages of caries. Here X-ray microtomography, optical, Raman, atomic force, scanning electron microscopy, and nanoindentation were simultaneously applied.

Magneto-electro-elastic coupling was studied in [10,11]. In [10], linear vibrations and buckling of nanoplates in a hygro-thermal environment were analyzed. To this end, a strain gradient nonlocal approach was used. Finally, using a variational approach nonlinear deformations of a nanobeam considering piezo- and flexomagneticity were studied in [11].

The content of the SI reflects the state of the art in the field of micro- and nanomechanics. It combines new theoretical models with modern experimental studies of materials.
